# Physiotherapists’ Perceptions and Willingness to Use Telerehabilitation in Greece: A Cross-Sectional Study

**DOI:** 10.7759/cureus.32317

**Published:** 2022-12-08

**Authors:** Maria Tsekoura, Konstantinos Fousekis, Sofia Lampropoulou, Sofia Xergia, Theofani Bania, Elias Tsepis, Evdokia Billis

**Affiliations:** 1 Physiotherapy Department, School of Health Rehabilitation Sciences, University of Patras, Patras, GRC

**Keywords:** covid 19, pandemic, perceptions, physiotherapists, telerehabilitation

## Abstract

Introduction

Coronavirus disease 2019 (COVID-19) has affected the healthcare system and the practice of physiotherapists. Telerehabilitation is an alternative method of delivering physiotherapy services. The aim of this study was to investigate physiotherapists’ knowledge, beliefs, and willingness to use telerehabilitation in Greece during the COVID-19 pandemic.

Materials and methods

In this cross-sectional study, Greek physiotherapists completed an online survey between January and February 2022. A questionnaire was distributed via the Panhellenic Physiotherapy Association (PSF). The questionnaire involved 26 items on demographic background, use of technology, overall perceptions, the experience of telerehabilitation, and their opinion on the future of telerehabilitation. The study protocol was approved by the Ethical Committee of the University of Patras, Greece.

Results

Participants in this study were 213 physiotherapists (female 57.7%; mean age 39.84±8 years). Most physiotherapists (n=118; 55.4%) were working in a private clinic in the areas of outpatient orthopedics, geriatrics, and neurorehabilitation. Overall, most participants (55%) reported increased use of telerehabilitation strategies during the COVID-19 pandemic. A total of 130 physiotherapists (n=61.3%) believed that telerehabilitation may be beneficial as a supplementary way of patient management. Greek physiotherapists made use of low-cost and easily accessible digital technologies, such as mobile phones and online meeting tools (e.g., Skype, Zoom). Although most physiotherapists (79.8%) reported that they wanted to receive more information about digital technology and telerehabilitation, only 42.1% of them did intend to work remotely after the pandemic.

Conclusion

Most of the participants were willing to deliver physiotherapy via telerehabilitation. Specific education and training programs need to be provided to physiotherapists during and after the pandemic. Healthcare managers should consider the use of telerehabilitation and design guidelines and policies to manage telerehabilitation practices in Greece.

## Introduction

Telehealth is the use of telecommunications and digital and virtual technology to deliver healthcare and patient rehabilitation [1]. Telerehabilitation (considered a branch of telehealth) refers to the delivery of rehabilitation using telecommunications technologies [2]. Telerehabilitation appears to have begun in the mid-late 1990s [3] and was developed to provide equitable access to people who are geographically remote and/or physically and economically disadvantaged [4]. The 2020 coronavirus disease 2019 (COVID-19) pandemic has changed many aspects of people’s lives [1] and affected traditional in-person rehabilitation care services worldwide [5]. Social distancing, the prolonged quarantine periods during the pandemic, and the fear of exposure to severe acute respiratory syndrome coronavirus 2 (SARS-CoV-2) required physiotherapists (PTs) to modify the way rehabilitation is delivered to patients [6]. It seemed an urgent need to adopt telerehabilitation in some healthcare settings [7] as an alternative method to usual face-to-face treatments [8]. Although telerehabilitation seems to have positive clinical results and can be a beneficial method of rehabilitation for various conditions (patients with cardiorespiratory, musculoskeletal, and neurological conditions) [9], there is a knowledge gap in the literature concerning physiotherapists’ (PTs) beliefs and willingness to use telerehabilitation during the COVID-19 pandemic in Greece. Thus, the aim of this study is to investigate the current knowledge, beliefs, and willingness of Greek PTs regarding the use of telerehabilitation.

The material contained within this manuscript was orally presented at the World Congress of Osteoporosis, Osteoarthritis, and Musculoskeletal Diseases on 24-26 March 2022 in Barcelona, Spain.

## Materials and methods

A nationwide cross‐sectional anonymous online survey was conducted among Greek PTs between January and February 2022. An original self‐administered questionnaire was developed by the study authors (Appendices). All questions were based on the review of the literature. Pretests were performed with four individuals for linguistic purposes and to establish face validity. The questionnaire involved 25 items divided into four parts: 1) demographic data, 2) technology usage, 3) overall perceptions/experience and challenges of telerehabilitation, and 4) willingness to use telerehabilitation. PTs eligible to participate in this study were 1) licensed by the Panhellenic Physiotherapy Association (PSF), 2) actively practicing physiotherapy in Greece, and 3) voluntary informed consent to participate in the study. This questionnaire was distributed via the PSF. Participation in this study was voluntary.

Ethics

The present study protocol was approved by the Ethical Committee of the University of Patras, Greece (approval number 12595).

Data analysis

Descriptive statistics were used to analyze the results. Frequencies and percentages were calculated for the participants’ demographic data. Statistical results were considered significant at the 5% critical level (p<0.05). Descriptive statistics and analysis of data were performed using IMB SPPS Statistics 28.0 (IBM Corp, Armonk, NY).

## Results

A total of 213 PTs participated in the study (n= 213; 90 male and 123 females).

Demographics

The participants had a mean age of 39.8 (SD=8) years and the majority of them were female (57.7%) (Table 1). Most PTs (n=118; 55.4%) were working in a private clinic, in the areas of outpatient orthopedics (n=151;74.4%), geriatrics (n=112; 55.2%), neurorehabilitation (n=105;51.7%), sports therapy (n=82;40.4%), cardiorespiratory rehabilitation (n=43;21.2%), and pediatrics rehabilitation (n=24;11.8%).

**Table 1:** Participants’ characteristics

Variable	Τotal participants (n=276)
Mean ± SD	
Αge (years)	39.84±8
Male	
Years of working	12±5.7
Number and percentage (%)	
Gender	
Female	123 (57.7%)
Male	90 (42.3%)
Level of education	
Bachelor	91 (52.4%)
MSc	85 (40.1%)
PhD	16 (7.5%)
Patient contact during the COVID-19 pandemic	203 (95.3%)
Main working area	
Private clinic	118 (55.4%)
Education	53 (24.9%)
Hospital	25 (11.7%)
Research	12 (5.6%)
Hours of working/week	
< 10	31 (25%)
10-20	21 (10.1%)
20-30	24 (11.5%)
30-40	56 (26.9%)
>40	6 (36.5%)

Technology usage

For personal purposes, almost all participants reported that they used digital tools daily (81.7%, n = 174), or three to five days per week (13.6%, n = 29). For personal reasons, most PTs use laptops/computers (n=190;89.2%), and/or phones (n=166;77.5%), and live streaming platforms (n=88;41.3%). For professional purposes, the majority of the participants stated that they use digital tools daily (n = 98, 46%), three to five days per week 24.4.% (n = 2) used digital tools daily, 25.8% and only one never or less than once per week. They used laptops/computers (n=178;83.6%), and/or mobile/phone devices (n=127;59.6%), live streaming platforms such as Zoom, Skype, etc. (n=101;47.4%).

Barriers to telerehabilitation practice

Greek PTs record the following as significant barriers: patient confidentiality, lack of knowledge, availability of equipment, and inadequate training.

Overall perceptions/experience and challenges of telerehabilitation: Overall, most participants (n=116; 55%) reported the increased use of telerehabilitation strategies during the COVID-19 pandemic. Out of 116 PTs, 52.9% started to practice some form of telerehabilitation during the COVID-19 pandemic (Table 2). In addition, 68.8% used technology for follow-up and the progress of the therapy. Eighty-seven PTs (69.6%) reported using the telephone, 65 (52%) used live streaming platforms, and 42% (n = 87) used laptops/computers. Seventy point seven percent (70.7%) of the participants who used some method of telerehabilitation did not record actions regarding the security of personal data.

**Table 2:** Use of telerehabilitation services before and during the lockdown (n = 119)

Physiotherapists providing telerehabilitation services	Number and percentages (%)
Before COVID-19 pandemic and lockdowns	55 (46.2%)
During COVID-19 pandemic and lockdowns	64 (53.7%)

Willingness to use telerehabilitation

Most PTs (n=130;61.3%) believed that telerehabilitation may be beneficial as a supplementary way of patient management. Greek PTs made use of low-cost and easily accessible digital technologies, such as mobile phones and online meeting tools (e.g., Skype, Zoom). Although most of them (n=170; 79.8%%) reported that they want to receive more information about digital technology and telerehabilitation, only 42.1% (n=48) of them did intend to work remotely after the pandemic.

## Discussion

The present study showed that most of the Greek PTs practiced some form of telerehabilitation during the COVID‐19 pandemic. The majority of Greek participants expressed a positive perception of this form of rehabilitation. This finding is similar to PTs working in Kuwait [1] and India [10]. Telerehabilitation offers benefits including a reduction in travel for the service user, access to telehealth physical therapy, and safety during the COVID-19 pandemic [1,9,11,12]. In the last few years, telemedicine applications have been increasing due to the development of advanced telemedical and computer devices [13]. In addition, there was a rapid and expansive rollout of telerehabilitation during the COVID-19 pandemic [11,12]. In countries, such as Australia [14], the USA [15], and Canada [16], the COVID-19 pandemic fostered the implementation of remote physiotherapy [8].

Telerehabilitation includes services such as assessment, diagnosis, prognosis, treatment, and follow-ups [17]. The majority of Greek PTs used it for follow-ups (68.8%). In a study conducted among PTs working in Ireland, the limited scope of the physical examination (86%) via telehealth is a significant disadvantage [11]. The main barriers recorded by Greek PTs were patient confidentiality, lack of knowledge and availability of equipment, and inadequate training. These barriers have also been recorded in literature [1,11-15]. Technology barriers are considered a major issue in implementing telerehabilitation services [16]. In Saudi Arabia, PTs reported a lack of technical and staff skills as the main barriers to telerehabilitation [17]. Identification of the barriers to the implementation of telerehabilitation systems could help eliminate them [1]. Specific barriers can be overcome through training [18,19]. Future research and medicolegal issues should be addressed. Healthcare decision-makers should design guidelines and policies [1] to manage telerehabilitation practices in Greece. Further, dialogue between clinicians and health care providers may provide beneficial results in timely collaborative practice based on current evidence and societal needs in health care [12,17]. Recently, World Confederation for Physical Therapy (WCPT) identified resources, including a tool kit for digital practice implementation, in order to help PTs worldwide [20].

Important issues are data security and patient confidentiality. In the present study, most PTs who used some method of telerehabilitation did not any action regarding the security of personal data. PTs should be concerned about data security, which seems to remain a disadvantage of digitalization [21-24]. Physiotherapeutic associations and societies have a significant role in providing orientation to address this issue [22,23].

The present study recorded that the majority of the Greek PTs were willing to use telerehabilitation in the future. This finding is consistent with previous studies [1,8,10,11]. One of the main results of the Irish study was that telehealth is a sustainable alternative mode of healthcare delivery [11]. A small percentage of Greek PTs do not have the will to receive more information about telerehabilitation and prefer to practice physiotherapy via conventional face-to-face methods. In a study conducted in Switzerland, the majority of participants stated that they did not intend to work remotely in the future [8]. A study among Belgian and French PTs was conducted between 17 January 2021 and 17 March 2021 (the second wave of the COVID-19 pandemic) investigating the perceived usefulness of telerehabilitation among 107 PTs and 68 patients concerned with musculoskeletal disorders. Although telerehabilitation has experienced a renewed interest because of the COVID-19 pandemic, a great majority of PTs working in Belgium and France (76%) had never used telerehabilitation at the time that they answered the questionnaire [25]. This may be due to the nature of the profession. Many methods and techniques require a physical presence and hands-on interventions [1,26-28]. PTs working in Sweden with people with neurological diseases or older adults had an overall positive perception of the use and willingness to learn about telerehabilitation [9,29]. Future studies should investigate the opinions of PTs in different fields of rehabilitation.

Regarding the effectiveness of telerehabilitation services, the results of a systematic review showed that telerehabilitation offers beneficial clinical results regarding physical function, especially in patients with neurological conditions. Future studies are required in order to investigate the effectiveness of telerehabilitation services [9], as telehealth is still in the early stage of implementation [11]. The success of telerehabilitation depends also on the digital competencies of both PTs and patients [8,26]. Future research should explore the needs and perceptions of Greek patients.

This study was the first to be conducted with Greek PTs regarding telerehabilitation practices. A strength of the survey was that it was supported by the PSF, which highlights the significance of the topic. However, the sample was small compared to the number of active licensed PTs in Greece, making it not representative [30]. Data regarding the actual number of PTs who received the questionnaire could not be collected; hence, information regarding non-responders and response rate could not be determined.

## Conclusions

This study highlights the importance of understanding PTs' perceptions of telerehabilitation in clinical practice. The present study identifies the willingness and the need for further education and training regarding telerehabilitation. The majority of Greek participants expressed a positive perception of telerehabilitation. In addition, Greek PTs want to receive more information about digital technology and continuous educational training regarding telerehabilitation practice. Researchers recommend communication between health policy, legislators, and health professionals in order to develop guidelines and manage telerehabilitation practices in Greece. Future research is needed regarding the lack of data security and knowledge on legal frameworks for telerehabilitation practices.

## Appendices

**Table 3:** Questionnaire

	Category of questions	Question	Values
	Sociodemographic		
		Gender	Male, Female, I don’t want to specify
1		Age (year)	
2		Work field	Hospital, Rehabilitation center, Home therapy, Teaching, Research, Other
3		Years of working	
4		Do you have contact with patients during your work as physiotherapist?	Yes /No
5	If YES Q4	What kind of patients do you mainly see working as physiotherapist?	Musculoskeletal, neurological, paediatrics, athletes, cardiorespiratory, geriatrics, other
6		How many effective working hours per week do you work?	<10, 10-20, 20-30, 30-40, >40
7		Level of education	Bachelor degree, MSc, Phd
8		Do you participate in educational lifelong courses?	Yes/No
	Attitude towards technology/knowledge		
9		Which digital tools do you use for personal purposes?	PC/Laptop/Tablet, Instagram/Twitter/Youtube, etc., Online Meeting tools (Zoom, Skype, MS Teams, etc.), Smart Watches, Smartphone applications, other
10		Which digital tools do you use for professional purposes?	PC/Laptop/Tablet, Instagram/Twitter/Youtube, etc., Online Meeting tools (Zoom, Skype, MS Teams, etc.), Smart Watches, Smartphone applications, other
11		How often do you use technology means for personal reasons?	Never 1-2 times per months Once per week 3-5 times per week Every day
12		How often do you use technology means for professional purposes?	Never 1-2 times per months Once per week 3-5 times per week Every day
	Overall perceptions/experience and challenges of telerehabilitation		
13		Do you offer tele/online- therapy?	Yes/No
14	If YES Q 13	Did you already offer tele/online- therapy BEFORE the lockdown?	Yes/No
15	If YES Q13	In which part of patient management of therapy did you use digital technology?	Examination and evaluation, Diagnosis, Provide the treatment, Patient education, Follow up, Improve the treatment adherence (e.g. sending reminder), Therapy monitoring (e.g. outcome assessment)
16	If YES Q13	Which tool did you use to perform tele/online therapy with your patients	Consultation hours, online, Phone, Viper/ WhatsApp, Messenger, Skype/Zoom, Other
17	If YES Q13	In which patient did you use tele/online therapy?	COVID-19 patient, musculoskeletal, neurological, respiratory, cardio, geriatrics, athlete, other
18	If YES Q13	What actions have you taken regarding data protection?	Consents form, No action, Other
19	If YES Q13	Do you charge for Tele/Online Therapy?	Yes / No
20		Will you keep offering tele/online therapy after Corona?	Yes / No
21		Do you think that telerehabilitation is beneficial for patients?	Yes / No
22	If NO Q11	Why did you not offer tele/online therapy	I was able to provide my patients with sufficient care in another way, I cannot observe the patient adequately, I miss the hands on experience, The technical possibilities are unknown to me or my patients, Other reason
23		Barriers for tele/online therapy?	Lack of knowledge regarding telerehabilitation Inadequate training, lack of equipment, patients confidentiality, other
	Willingness to use telerehabilitation?		
24		Would you like to have more information/a training offer regarding Tele/Online Therapy?	Yes/No
25		What kind of information/support/training toy think would be useful?	Knowledge about infrastructure, Knowledge about applications, Knowledge about law and data protection, Knowledge about therapy cost, Knowledge about effectiveness, other
